# Extramedullary sudden blast crisis in chronic-phase chronic myeloid leukemia during first-line treatment with nilotinib

**DOI:** 10.1038/bcj.2016.66

**Published:** 2016-08-26

**Authors:** M K Angelopoulou, J V Asimakopoulos, Z Galani, G Levidou, M Roumelioti, T P Vassilakopoulos, P Korkolopoulou, P Panayiotidis

**Affiliations:** 1 Department of Hematology and BMT Unit, National and Kapodistrian University of Athens, School of Medicine, Laikon General Hospital, Athens, Greece; 2 1st Department of Pathology, National and Kapodistrian University of Athens, School of Medicine, Laikon General Hospital, Athens, Greece; 3 Molecular Hematology Laboratory, 1st Department of Propaedeutic Medicine, National and Kapodistrian University of Athens, School of Medicine, Laikon General Hospital, Athens, Greece

The natural course of chronic myelogenous leukemia (CML) has changed dramatically since the introduction of tyrosine kinase inhibitors (TKIs) with >90% of the patients achieving long-term disease control. Development of resistance with progression to accelerated phase/blastic crisis (AP/BC) is observed in a limited percentage of patients^1^ and is mainly documented in those who do not respond ‘optimally' according to cytogenetic and molecular response criteria at given intervals.^1, 2^ Rare reports of an abrupt blastic transformation defined as sudden blast crisis (SBC) have been described in chronic-phase (CP) CML patients, who had already achieved optimal response, that is, complete cytogenetic (cCgR) or even deep molecular response (MR^4^ or better).^2, 3, 4^ Herein, we present an extremely rare case of unpredictable blastic transformation in a patient receiving nilotinib.

A 53-year-old Caucasian female patient was diagnosed with CP CML. At diagnosis, the spleen was palpable 1 cm below left costal margin, hemoglobin was 11.3 g/dl, platelet counts were normal and white blood cell counts were 105 × 10^9^/l with a left shift, 12% basophils and 5% blasts. Nested reverse transcriptase PCR from peripheral blood (PB) revealed the presence of the chimeric bcr-abl1 (β3α2 transcript), whereas bone marrow (BM) cytogenetics confirmed the presence of t(9;22)(q34;q11) translocation in all metaphases. According to Sokal index and Hasford score, the patient was classified as high risk (1.20 and 1759, respectively).^5^ However, based on the newer European Treatment and Outcome Study risk score, her disease was stratified as low risk (score <87).

She was started on nilotinib 300 mg bid and complete hematologic response (cHR) was documented within 30 days after the initiation of nilotinib. At 3 months on nilotinib treatment, she achieved a cCgR, whereas quantitative real-time PCR (RQ-PCR) revealed a bcr-abl/abl ratio of 0.07%. At her following reevaluation (6 months after nilotinib), the patient remained in cHR and cCgR with a bcr-abl/abl ratio of 0.05%. Thus, our patient was considered as an optimal responder, according to the current European Leukemia Net (ELN) recommendations for CML.^5^

Seven months after diagnosis and 1 month after the documentation of optimal response, while receiving nilotinib, the patient complained of right lower quadrant pain, loss of appetite and weight, without significant findings on clinical examination. An abdominal computed tomography scan revealed right iliac lymphadenopathy of 4 cm in maximal diameter. Shortly, thereafter, she developed right inguinal lymphadenopathy. A lymph node biopsy was performed and lymph node cells were also isolated. Lymph node imprints showed immature blast cells, whereas fluorescence *in situ* hybridization analysis showed the presence of the bcr-abl1 fusion gene in 81% of 200 interphase nuclei. The histologic examination of the lymph node demonstrated infiltration by an immature cell population, with fine-shaped nuclear chromatin and prominent nucleoli with a high mitotic and apoptotic rate (Figure 1). Immunochemistry was positive for MPO (diffuse), c-kit, CD34, CD56 and CD61 and negative for neuroendocrine markers. Cytogenetic analysis of the lymph node cells showed 100% positivity for Ph chromosome with an additional tetrasomy of chromosome 19; *48,XX,t(9;22)(q34;q11),+19,+19[25]*. The bcr-abl/abl ratio in the lymph node cells was 24%. Mutation analysis by direct sequencing showed the presence of the T315I. At the same time, the patient remained in cHR, while trephine biopsy and BM cytogenetics were normal and bcr-abl/abl ratio in peripheral blood was 0.06%. Thus, the patient still met the criteria of optimal response according to the ELN guidelines when peripheral blood was examined,^5^ but the presence of extramedullary blastic transformation, classifies her as a CML case with blastic transformation.

Nilotinib was stopped, and induction chemotherapy for acute myeloid leukemia with the *‘7+3'cytarabine-idarubicin* regimen was administered. Ponatinib was not available at that time and her siblings were not histocompatibility leukocyte antigen-compatible to her. She did not respond to chemotherapy and she developed new abdominal lymph node enlargement. She finally developed BM disease and expired due to sepsis while receiving salvage second-line chemotherapy.

BC of CML is defined as the presence of 20% PB or BM blasts or presence of extramedullary infiltration, as per the World Health Organization classification and categorized as lymphoid or myeloid by immunohistochemistry and flow cytometry.^2^

The introduction of TKIs has revolutionized the treatment of CML by changing the natural course of the disease, offering rapid and durable responses and making CML the first malignancy with a life expectancy similar to that of the general population. The risk of progression to AP/BC is 0–3% per year during the initial 6 years and is correlated with risk stratification scores.^1^ The majority of these events can be predicted on the basis of individual cytogenetic and molecular responses. Thus, patients who fail to achieve at least a partial cytogenetic response at 6 months on imatinib have a 15–20% probability of progression to AC/BC.^1^ Second-generation TKI nilotinib is associated with a significantly decreased incidence of progression to AP/BC, during the initial 3 years of treatment (0.7% on nilotinib vs 4.8% on imatinib).^6^

The majority of patients who progress to BP are the ones who show suboptimal responses to TKIs at predefined time points.^1^ On the contrary SBC refers to the abrupt onset of BC, defined as the unexpected development of BC despite a documented ‘optimal' response to TKIs in the immediately preceding BM analysis according to the ELN Recommendations,^5^ and within 3 months of a normal complete blood count.^2, 3, 4^ Most impressively, it is observed even after deep molecular response—the most reliable surrogate end point of long-term disease control. SBC is rare and has been reported at a rate of 2.2% with interferon and in 0.7–5.9% of imatinib-treated patients,^2, 3, 4^ while to the best of our knowledge, there are no reports of SBC during nilotinib treatment. SBC cases differ from those who develop AP/BC as a result of imatinib failure: SBC is associated with low- or intermediate-risk Sokal scores, short median time between imatinib initiation/optimal response and SBC (median: 7–9 months),^2, 4^ predominance of lymphoid phenotype, high incidence of clonal evolution and dismal outcome.^4^ In the few cases, where mutational analysis was performed, the frequency of abl mutations was low.^2, 3, 4^

The present case corresponds to an isolated extramedullary SBC under nilotinib treatment in a patient who fulfilled all the criteria of optimal response. The novelty of this case relies on the fact that there are only 10 cases of isolated extramedullary SBC reported so far and only 1 involving the lymph nodes.^7, 8, 9, 10, 11, 12, 13, 14, 15^ Moreover, this is the first case of SBC reported so far under nilotinib first-line treatment. In addition, this is among the very few cases, where mutational analysis was undertaken from the affected tissue, and the only one in which a pan-resistant T315I mutation was documented.

Most reports and series of patients referring to SBC involve cases with BM SBC.^2, 3, 4^ Isolated extramedullary SBC is extremely rare and may involve central nervous system, skin, soft tissue, breast, bones, gastrointestinal, genitourinary tract and lymph nodes. Table 1 summarizes disease characteristics and outcomes in reported cases of CP CML with extramedullary SBC on first-line treatment with imatinib.^7, 8, 9, 10, 11, 12, 13, 14, 15^ Interestingly, a male predominance is noticed and 7/10 cases are <60 years. In contrast to the short interval between imatinib initiation and SBC described in SBC involving the BM, only 3/10 extramedullary SBC reported patients had received imatinib for less than a year up to SBC. Our case, however, developed SBC early in the course of her treatment (at 7 months) and only 1 month after the documentation of an ongoing cCgR. Central nervous system is the most common site of localization, probably due to poor drug penetration across the blood–brain barrier. Clonal evolution is reported in 4/5 patients in whom cytogenetics from the affected tissue were performed, whereas myeloid phenotype seems to be more common. No mutations in abl were present in the single case tested, whereas ours tested positive for the T315I mutation. It is a matter of question whether the resistant T315I clone preexisted or developed during treatment.

Although multiple studies have addressed TKIs resistance, the exact underlying pathogenetic mechanisms for SBC remain rather obscure. The frequent presence of clonal evolution suggests that some aggressive sub-clones of primitive Ph+ cells may survive, despite TKI treatment. These cells may expand exponentially due to a proliferative advantage over normal hematopoiesis. ABL mutations are not frequently reported in SBC. However, in our case the T315I mutation was documented, explaining the resistance of these cells to TKI treatment. In addition, the Ph chromosomal amplification in the lymph node cells offers an additional cause for resistance to TKI therapy.

In conclusion, we presented the first case of extramedullary SBC in a patient with CP CML during first-line treatment with nilotinib. Despite advances in molecular disease detection, more sensitive and effective methods of monitoring TKIs bioavailability and disease progression are still needed, considering the very poor prognosis of these patients. Moreover, organ-specific symptoms—initially thought to be unrelated to CML—in responding patients should not be overlooked, as they might imply extramedullary disease infiltration.

## Figures and Tables

**Figure 1:**
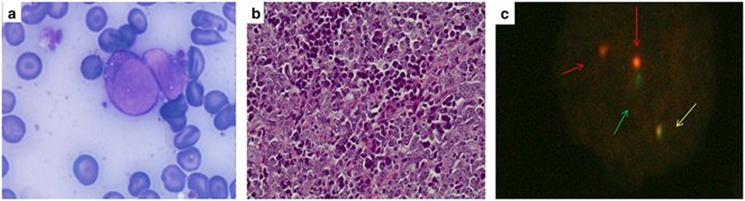
**(a)** May-Grünwald-Giemsa staining of the lymph node smear showing two blasts characterized by an oval-shaped nucleus with a fine chromatin pattern and four well visible nucleoli and a slightly basophilic cytoplasm with small vacuoles and a high nuclear/cytoplasmic ratio. (**b)** Hematoxylin and Eosin stains (lymph node biopsy) showing the presence of medium- to large-sized blasts with fine nuclear chromatin and focally prominent nucleoli. (**c)** Representative photograph of a nucleus carrying typical M-BCR-ABL, t(9;22) translocation. Interphase fluorescent *in situ* hybridization was performed on lymph node touch preparations using LSI BCR/ABL ES Dual Color Translocation Probe (Vysis) (Abbott Laboratories, Abbott Park, IL, USA). The probe is hybridized to a nucleus showing one green signal (BCR), one large red signal (ABL), one smaller red signal (residual ABL) and one fused red and green signal perceived as yellow (BCR-ABL fusion).

**Table 1:** Summary of reported CP CML cases with isolated extramedullary SBC during first-line treatment with IM in the literature

*Patient#/ref*	*Age/gender*	*Time from IM to SBC(months)*	*Optimal response, time to best response (months)*	*Time from optimal response to SBC (months)*	*Symptom/site*	*Tissue karyotype*	*BC lineage*	*BM karyotype/ blood bcr-abl%*	*Treatment/outcome*
Rajappa^7^	39/M	17	cCgR, NR	NR	Headache, vomiting/ CNS	NR	NR	cCgR/NR	ITCh/alive
Matsuda^8^	17/M	4	cCgR, 4	1	Headache/CNS	48,XY,+8,t(9;22),+10	Myeloid/B-lymphoid	cCgR/decreasing	ITCh+RT/CR→IM
		11	cCgR, NR	NR	Subcutaneous mass/ right neck	47 XY,+6, t(9;22) [15%] 46, XY, t(9;22) [40%]	Myeloid/T-lymphoid	cCgR/NR	Increase IM dose (600 mg)+RT/PD
Simpson^9^	62/F	14	cCgR, 12	2	Bowel obstruction/small intestine	Bcr/abl+	Myeloid	cCgR/NR	Increase IM dose (800 mg)+RT/death
Simpson^9,^ a	54/M	24	cCgR, 6	18	Skin lesions/skin	Bcr/abl+	Myeloid	cCgR/NR	SysCh/alive
Barlow^10^	68/M	20	cCgR, 6	14	Headache, imbalance, tremor and leg cramps/CNS	45–46, X, -Y, der(1)t(1;8), i(9), t(9;22), i(17)	B-lymphoid	NR/ongoing MaMolR	ITCh+RT+DAS
Kyung-Woo^11^	39/M	37	cCgR, NR	10	Headache, diplopia/CNS	NR	Lymphoid	NR	ITCh
Shune^12^	50/F	6	MaMolR, 5	1	Back pain/lymphadenopathy	NR	Myeloid	NR/No abl mutations	SysCh/alive
Park^13^	54/M	7	MaCgR, 6	1	Headache/CNS	Bcr/abl +	B-lymphoid	MaCgR/NR	ITCh+RT/alive
Gomez^14^	33/M	~60	cCgR, 4	56	Fever, headache, nausea and confusion/ CNS	46,XY,t(9;22)	NR	cCgR/cMolR	ITCh+DAS/alive-severe visual deficit
Gulati^15^	66/M	36	MaMolR,3	33	Ocular pain, loss of vision/ocular	49,XY,+8,+9,+22, t(9;22)	Myeloid	cCgR/cMolR	InCh+ITCh+DAS+Allo-SCT/alive

Abbreviations: Allo-SCT, allogeneic stem cell transplantation; BC, blastic crisis; BM, bone marrow; cCgR, complete cytogenetic remission; CML, chronic myeloid leukemia; cMolR, complete molecular response; CNS, central nervous system; CP, chronic phase; CR, complete remission; DAS, dasatinib; F, female; IM, imatinib; InCh, induction chemotherapy; ITCh, intrathecal chemotherapy; M, male, MaCgR, major cytogenetic response; MaMolR, major molecular response; NR, not reported; PD, progressive disease; RT, radiotherapy; SBC, sudden blast crisis; SysCh, systemic chemotherapy.

a Prior treatment with interferon-α as a CP CML.
